# Analytical and experimental study of a valveless piezoelectric micropump with high flowrate and pressure load

**DOI:** 10.1038/s41378-023-00547-7

**Published:** 2023-06-05

**Authors:** Jiafeng Ni, Weipeng Xuan, Yilin Li, Jinkai Chen, Wenjun Li, Zhen Cao, Shurong Dong, Hao Jin, Lingling Sun, Jikui Luo

**Affiliations:** 1 Ministry of Education Key Lab. of RF Circuits and Systems, College of Electron. & Info., Hangzhou Dianzi University, Hangzhou, 310018 China; 2 Zhejiang Key Laboratory of Large-Scale Integrated Circuit Design, Hangzhou Dianzi University, Hangzhou, 310018 China; 3 Key Lab. of Adv. Micro/Nano Electron. Dev. & Smart Sys. of Zhejiang, College of Info. Sci. & Electronic Eng., Zhejiang University, Hangzhou, 310027 China; 4 International Joint Innovation Center, Zhejiang University, Haining, 314400 China

**Keywords:** Electrical and electronic engineering, Electronic devices

## Abstract

Miniaturized gas pumps based on electromagnetic effect have been intensively studied and widely applied in industries. However, the electromagnetic effect-based gas pumps normally have large sizes, high levels of noises and high power consumption, thus they are not suitable for wearable/portable applications. Herein, we propose a high-flowrate and high-pressure load valveless piezoelectric micropump with dimensions of 16 mm*16 mm*5 mm. The working frequency, vibration mode and displacement of the piezoelectric actuator, the velocity of gas flow, and the volume flowrate of the micropump are analyzed using the finite element analysis method. The maximum vibration amplitude of the piezoelectric actuator reaches ~29.4 μm. The output gas flowrate of the pump is approximately 135 mL/min, and the maximum output pressure exceeds 40 kPa. Then, a prototype of the piezoelectric micropump is fabricated. Results show that performance of the micropump is highly consistent with the numerical analysis with a high flowrate and pressure load, demonstrated its great potential for wearable/portable applications, especially for blood pressure monitoring.

## Introduction

Driven by strong application prospects in various fields, including medicine, health care^1,2^, microfluidics^3^, and microelectronics circuit cooling^4^, piezoelectric (PE) micropumps using piezoelectric materials as the driving components have been extensively explored and developed. PE micropumps have small sizes, good integrability and low power consumption levels^5,6^, and they have become a popular research topic in industry and academia^7^. The continuous development of MEMS technology has led to new strategies for designing PE micropumps^8^, and many kinds of structures have been developed, such as pumps with triple vibrators^9^ and pumps with direct spray structures^10^.

Based on their working principles and structures^11^, PE micropumps can be classified into piezoelectric peristaltic pumps, valve-based PE pumps, and valveless PE pumps^12,13^. The PE peristaltic pumps have complex structures and rely on multiple vibrators to realize the creep effect in the flow direction. The valve-based PE pumps can be further divided into active valve pumps and passive valve pumps based on the types of valves used. Although valve-based PE pumps have a positive effect on controlling the fluid movement direction, there is a phase difference between the opening and closing of the valves and the vibration of the PE actuators. The hysteresis becomes severer when the pump works at relatively high frequencies, resulting in unstable performance. Therefore, the valve-based PE pumps are only suitable for low-frequency drivers (less than 1 kHz)^14^. In addition, the valves gradually lose their function due to aging and fatigue, resulting in relatively short durability and poor reliability^15^. The structures of valveless PE pumps are much simpler than those of valve-based PE pumps. There are several approaches to realize PE-based valveless pumping^16,17^. One strategy is to utilize the synthetic jet effect to manipulate the flow resistance difference of the tube wall to generate a flow^18^. The other strategy is to utilize the change in the height ratio of the upper and lower chambers to induce a pressure difference between the inlet and outlet^19^. As there is no frictional wear or sticking effect, valveless pumps have long durability and good reliability. Their working frequencies can be significantly increased, even above 22 kHz, which is the upper-frequency limit of sounds that humans can hear. Therefore, valveless PE pumps have low operation noises, which is particularly important for wearable/portable applications. To increase the flowrates of PE pumps, pumps with multichambers in series or parallel connections have been investigated and explored^20^. Wang et al. reported a PE gas pump that can achieve a pressure load of 12 kPa^21^. However, PE pumps with a relatively high-pressure load is yet to be developed.

Herein, we propose a valveless PE micropump with a high flowrate and pressure load. The principle and structure of the PE micropump are analyzed numerically, and piezoelectric material selection is considered. We establish a three-dimensional (3D) model of the PE micropump and a finite element method (FEM) model of the PE actuator. The 3D bidirectional fluid–structure coupling analysis of the PE micropump and the optimization of the structural parameters of the micropump are demonstrated. Then, a prototype micropump is successfully fabricated, and its output flowrate and pressure load performance are investigated. The results show that the valveless PE pump can deliver a gas flowrate of ~144 mL/min and a pressure load of ~40 kPa, demonstrating excellent application potential for wearable blood pressure measurement devices.

## Analysis of the piezoelectric actuator and micropump

### Mechanical structure and operating principle

**Fig. 1:**
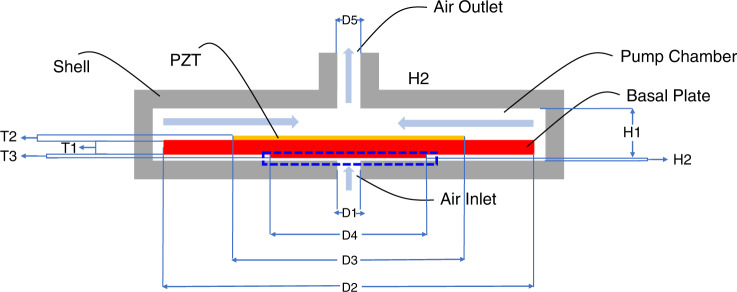
Schematic of the PE micropump. Cross-sectional schematic structure of the PE micropump

Figure 1 shows the schematic structure of the valveless PE micropump. The micropump is composed of an upper chamber with an air outlet, a PE actuator, and a lower thin chamber with an air inlet. The PE actuator vibrates up and down under a sinusoidal electrical signal. The vibration of the PE actuator and the bulge (blue box in Fig. 1) leads to a gas flow velocity and pressure change in the narrow gap. The pressure difference between the narrow gap and inlet/outlet induces gas flow. When the center of the PE actuator moves upward, the gas in the upper chamber is squeezed out from the air outlet, and there is an increased space between the bulge and the air inlet. The space increase induces a decrease in pressure in the gap; therefore, the gas in the atmosphere flows in from the inlet. When the center of the PE actuator moves downward, the gas at the air outlet is sucked in to form a backflow, the bugle compresses the space between the shell and the bugle, and the gas flows out of the inlet. However, due to the narrow gap structure in the lower chamber, the gas flow out of the inlet is less than that in the inlet.

### Design and analysis of the piezoelectric actuator

**Fig. 2:**
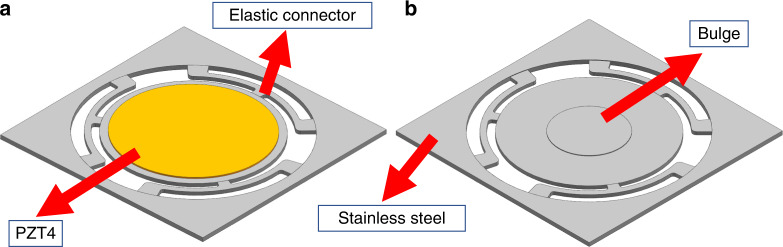
Structure of the PE actuator. **a** Top side **b** Bottom side

The piezoelectric actuator is the key component of PE micropumps, and its vibration characteristics determine the performance. Therefore, it is necessary to analyze the vibration mode and resonance mode of the PE actuator. The structure of the proposed PE actuator is shown in Fig. 2. The actuator is composed of a piezoelectric ceramic plate and an elastic basal plate. The yellow part in the middle is the piezoelectric ceramic lead zirconate titanate (PZT4), and the gray part is the stainless steel 304 basal plate. The dimensions and material parameters of the PZT4 specimen are summarized in Table 1.

**Table 1:** Dimensions and material parameters of PZT4

Parameter Symbol Unit			${\bf{PZT}}{\bf{4}}$
Thickness	T	$({mm})$	0.1
Diameter	D	$({mm})$	10
Density	$\,\rho$	$({10}^{3}{kg}/{m}^{3})$	7.5
Elastic constants	$s11$	$({10}^{-12}{m}^{2}/N)$	12.3
	$s12$		-4.1
	$s13$		-5.3
	$s33$		15.5
	$s44$		39
Piezoelectric constants	${d}_{31}$	$({10}^{-12}C/N)$	-123
	${d}_{33}$		289
	${d}_{15}$		496
Relative permittivity	$\varepsilon 11$		1475
	$\varepsilon 33$		1300

A circular bulge is designed in the center of the backside of the basal plate, with a height of 50 μm and a diameter of 5 mm. The bulge of the actuator membrane faces the thin fluid chamber, which has an air intake hole. During the vibration of the PE actuator, the circular bulge continuously impacts the thin fluid layer, inducing repeated pressure fluctuations between the thin fluid layer and intake hole, leading to a net gas volume flow in the inlet. The details will be discussed in the Analysis of Fluid–Structure Interaction section.

The flexure connection structures between the central plate and the fixed surrounding part determine the working frequency of the actuator. The width and thickness of the flexure connection structures are optimized to obtain a working frequency just above 22 kHz, which is higher than the maximum sound frequency that humans can hear^22^. However, the working frequency should not be too high, as the loss from vibrations increases with increasing vibration frequency. The flexure connections are the springs for the basal plate; they can reduce the stress of the basal plate and improve its displacement amplitude.

**Fig. 3:**
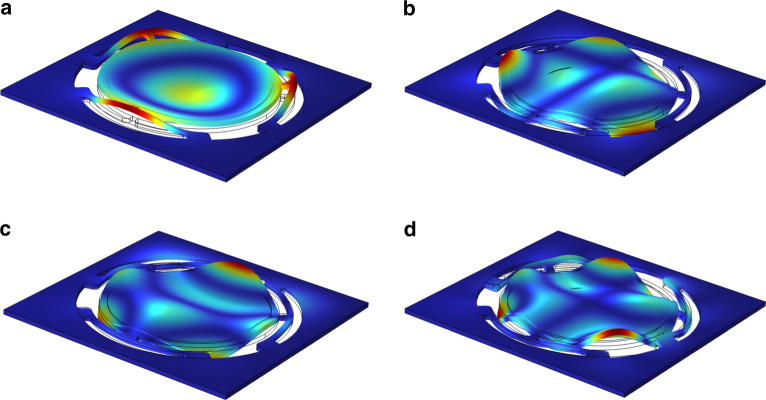
First four vibration modes of the PE actuator. **a** First-order. **b** Second-order. **c** Third-order. **d** Fourth-order

The displacement of the PE actuator is at a maximum when it is driven at its resonance mode, which is the frequency of the minimum impedance of the actuator. When the PE actuator is driven at different frequencies, the vibration modes are different. COMSOL Multiphysics (COMSOL 6.0) software is used to analyze the vibration characteristics. There are four resonant modes for the PE actuator, as shown in Fig. 3. The frequencies corresponding to all modes are summarized in Table 2.

**Table 2:** First four resonant frequencies of the PE actuator

Order	Resonant frequency/Hz
I	23,642
II	54,422
III	54,454
IV	86,319

**Fig. 4:**
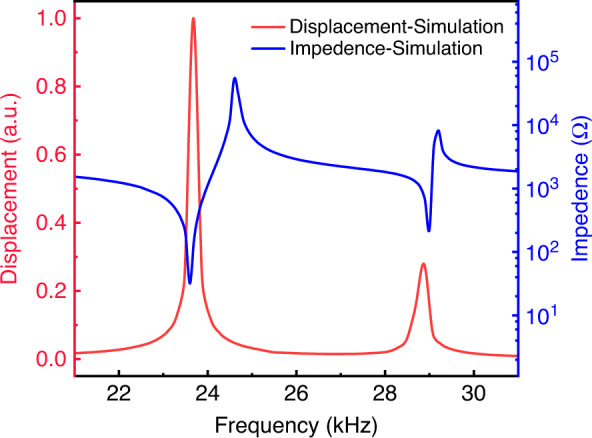
Impedance and displacement of the PE actuator. The impedance and displacement as a function of frequency for the PE actuator

As shown in Fig. 3a, when the PE actuator works at its first-order resonant frequency, the displacement of the PE actuator is the largest at the center. This vibration shape is particularly suitable for the design of micropumps because it provides the largest change in volume; thus, it has a high pumping efficiency. If the actuator works in other modes, the PE actuator has irregular torsional motions, resulting in poor output performance and low pumping efficiency. Therefore, these modes are not suitable for the design of micropumps. Figure 4 shows the impedance and displacement at different frequencies of the PE actuator. The actuator delivers the maximal displacement when it works at the first-order resonant frequency.

### Design and analysis of the piezoelectric micropump

**Fig. 5:**
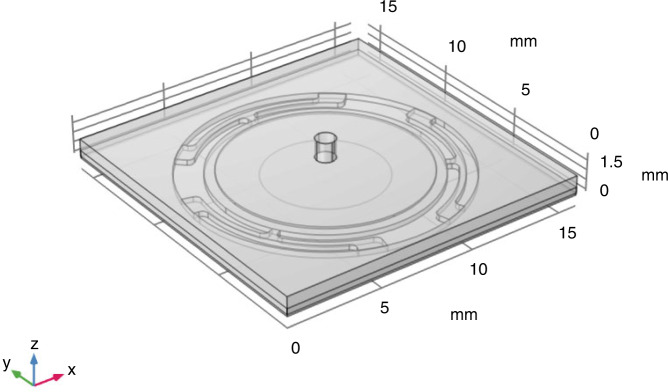
3D simulation model of the PE micropump. The 3D schematic structure of the PE micropump

The 3D simulation model with the parameters shown in Table 3 and labeled in Fig. 1 is shown in Fig. 5. The simplified 3D model includes a double-layer structure and a fluid domain with fixed boundaries. The double-layer structure is composed of a basal plate of stainless steel 304 and a piezoelectric layer of PZT4. The bonding layer between the stainless steel 304 and PE layers and the Ag electrodes on the upper and lower surfaces of the PZT is ignored here as it is much thinner than the layers of the other structures.

**Table 3:** Parameters of the micropump

Parameters	Symbol	Origin value (mm)
Diameter of Air Inlet	*D*1	1
Thickness of Basal Plate	*T*1	0.3
Length of Basal Plate	*D*2	16
Thickness of PZT	*T*2	0.1
Diameter of PZT	*D*3	10
Thickness of Bulge	*T*3	0.05
Diameter of Bulge	*D*4	5
Diameter of Air Outlet	*D*5	1
Height of Upper Chamber	*H*1	1
Height of Lower Chamber	*H*2	0.05

The simulation based on the 3D structural model has higher accuracy than that based on a 2D model. However, the 3D model-based simulation consumes considerable computing memory and time. To reduce the computing resource for the 3D model-based simulation, the 3D YZ axis symmetry is adopted to simplify the structure for simulation. This method reduces the solution degree of freedom of the entire model by 40% and minimizes the computing time without reducing the simulation resolution and accuracy.

Due to the involvement of fluid mechanics, there are great requirements and restrictions on the grid. Therefore, we use dynamic grids and boundary layer grids for the simulation. In particular, the shell is omitted in the model; therefore, the simulation is carried out with the prescribed mesh displacement set to zero at the outer boundary of the fluid domain. The boundary conditions of the inlet and outlet are set to a pressure of zero. The number of meshes of this model is approximately 2.5 million, and the instantaneous calculation requires approximately 48 hours (2 Intel(R) Xeon(R) Platinum 8358 P CPU @ 2.60 GHz 32-core processor, 256 GB memory) to reach a steady state for 200 cycles. The obtained average element quality of the mesh for the model is approximately 0.73, and the relative tolerance of the transient study is set as 0.1. The upper surface of the PZT is set to apply a sine wave excitation electrical signal with an amplitude of Vin, and the lower surface is set as the ground with fixed constraints for the surrounding square surface of the PE actuator.

### Analysis of fluid–structure interactions

To demonstrate the micropump output gas capability, a fluid–structure interaction analysis in the pump chamber is carried out. One of the elements used to determine whether the gas flow is laminar or turbulent is the Reynolds number (Re)^23^. Based on the definition of turbulence, there is a critical value of the Re number (approximately 2300 ~ 2800)^24^; if the Re number is greater than the critical value, the flow is turbulent, and the trajectory of fluid motion is irregular and disordered with time. Therefore, it is very important to evaluate the states of fluid motion and select the correct fluid interface. The Reynolds number is calculated by Eq. (1).

1 $$\mathrm{Re}=\frac{{\rm{\rho }}{UL}}{u}$$

where $U$, ${\rm{\rho }}$, and $u$ are the velocity, density, and viscosity coefficient of the air, respectively, and L is the diameter of the flow channel. The parameter values are as follows: $\rho =1.29{{kg}/m}^{3}$; $u=1.87* {10}^{-5}\,{Pa}/s$; and $L=1{mm}$. Through the simulation, the maximum velocity in the air domain is 46.7 m/s, and the obtained Re value is 3321.5, which is larger than the critical value of turbulent flow. However, many small eddies and disordered streamline movements appear in the fluid domain. Therefore, based on the obtained Re number and fluid flow phenomena, to precisely analyze the performance of the pump, the turbulent configuration is set to the air domain.

**Fig. 6:**
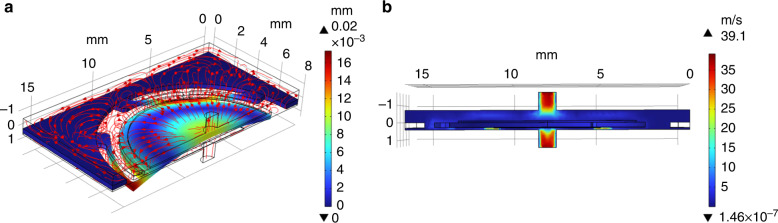
Simulation analysis of the gas domain in the micropump. **a** Streamline field. **b** velocity field distribution

Figure 6a shows the gas flow streamline field, which exhibits the gas flow direction distribution of the pump. Figure 6b shows the air velocity field distribution in the chamber of the micropump obtained by the FEM analysis. To precisely analyze the performance of the pump, the physical interface of the k-epsilon (k-ε) turbulent module (a two-equation model that gives a general description of turbulence through two transport equations (PDEs))^25^ is applied to the air domain in the pump, with the turbulent intensity set to medium (0.05). Additionally, a compressible flow (Ma < 0.3) is applied to the air domain, and the unspecified parameters are given the default settings.

The instantaneous air volume flowrate, Q_out_, can be obtained by integrating the fluid flow at the outlet with time, and it is expressed by Eq. (2).

2 $${Q}_{{out}}=\frac{{\int }_{{t}_{0}}^{{t}_{1}}\pi * {r}^{2}* v(r){dt}}{{t}_{1}-{t}_{0}}\,({mL}/\min )$$

**Fig. 7:**
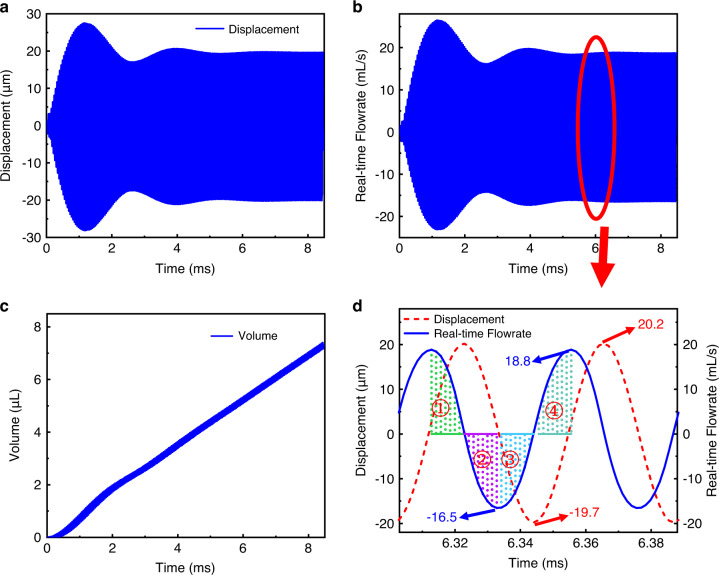
Simulation analysis of the micropump. Displacement of the central point of the PE actuator. **b** Real-time gas flowrate in the outlet. **c** Gas volume output with time. **d** Detailed real-time gas flowrate at the outlet and displacement of the PE actuator for two cycles

Where $v$ is the superficial gas velocity at the outlet of the pump in $m/s$ and $r$ is the radius of the gas outlet. Figure 7a illustrates the displacement evolution with time when the PE actuator is excited with a sinusoidal electrical signal. The displacement in the center of the PE actuator increases from zero to a maximum value of 29 μm in approximately 1.5 ms and then stabilizes to a value of approximately 20 μm, with an applied signal of 40 V and a frequency of 23.642 kHz. The instantaneous volume flowrate at the outlet is shown in Fig. 7b. A positive flow at the outlet suggests that the gas flows out of the chamber from the outlet, and vice versa for a negative flow. The gas volume output, V_out_, of the micropump with time can be calculated by integrating the outlet flowrate with time, and it is given by Eq. (3).

3 $$\triangle {V}_{{out}}={{V}_{{out}}}_{t=t1}-{{V}_{{out}}}_{t=t0}\,={\int }_{{t}_{0}}^{{t}_{1}}{Q}_{{out}}{dt}\,({mL})$$

The volume of the pumped-out gas by the micropump in 200 cycles is shown in Fig. 7c. The gas flowrate of the pump is calculated to be approximately 52 mL/min.

**Fig. 8:**
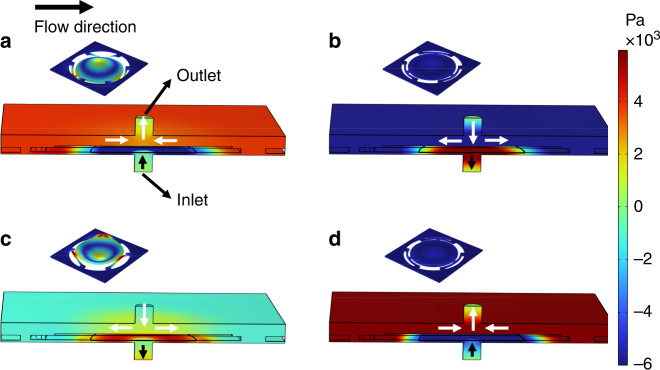
Pressure distribution and gas flow direction in the micropump chamber within one cycle. **a** t = T/4. **b** t = T/2. **c** t = 3 T/4. and **d** t = T

Figure 7d shows the real-time gas flowrate of the pump at the outlet and the displacement of the PE actuator. When the gas flowrate is negative, there is a backflow in the outlet, and the gas flows into the outlet. A positive gas flow suggests that the gas flows out of the outlet, and vice versa for a negative flow. Figure 8 shows the pressure distribution and gas flow direction in the micropump chamber in one cycle. Based on the figures, the working process of the pump can be explained as follows. One working cycle can be divided into four stages, where t = 0 is the time when the PE actuator is at zero displacement and is starting to move upward. In the first quarter cycle of the vibration, the PE actuator vibrates from the zero-displacement state upward and reaches the positive maximum position. The gas is pumped out of the outlet. Then, the instantaneous gas volume flowrate decreases from the maximum of 18.8 mL/min to 0 mL/min. For the second quarter of the cycle, the actuator vibrates downward from the positive maximum position, and the gas pressure in the upper chamber is lower than that in the outlet. The gas is sucked in from the outlet, as shown in Fig. 8b, and the gas volume flowrate from the outlet changes from 0 mL/min to −16.5 mL/min. In the third quarter of the cycle, the actuator vibrates upward, the displacement decreases from the zero-displacement to the minus maximum position, and the gas volume flowrate increases from −16.5 mL/min to 0 mL/min. In the last quarter of the cycle, the actuator vibrates upward from the minus maximum position to zero displacement, and the gas pressure in the upper chamber changes from a state lower than the outlet to a state higher than the outlet, pushing the gas out of the outlet, and the gas volume flowrate out of the outlet changes from 0 mL/min to 18.8 mL/min. In one cycle of operation, the gas pumped out of the outlet is much larger than the gas intake from the outlet. Therefore, for one vibration cycle, the net gas volume flow out of the outlet is positive. The reason for this phenomenon is that the heights below the actuator and above the actuator are different. According to Fig. 7d, when the actuator moves from the maximum positive position to the maximum negative position, the real-time gas flowrate from the outlet changes from positive to negative. During the downward movement of the actuator, the height of the lower chamber below the actuator is much smaller than that of the upper chamber above the actuator. The narrow space of the lower chamber induces a relatively higher pressure. When the actuator moves upward, the height of the upper chamber above the actuator is relatively high, which induces a lower pressure than that of the lower chamber below the actuator when the actuator moves downward. Therefore, in one cycle, the gas volume flowing out and in of the outlet is asymmetric, and a positive net gas flowing out of the outlet is obtained^26^.

## Structural optimization of the piezoelectric micropump

**Fig. 9:**
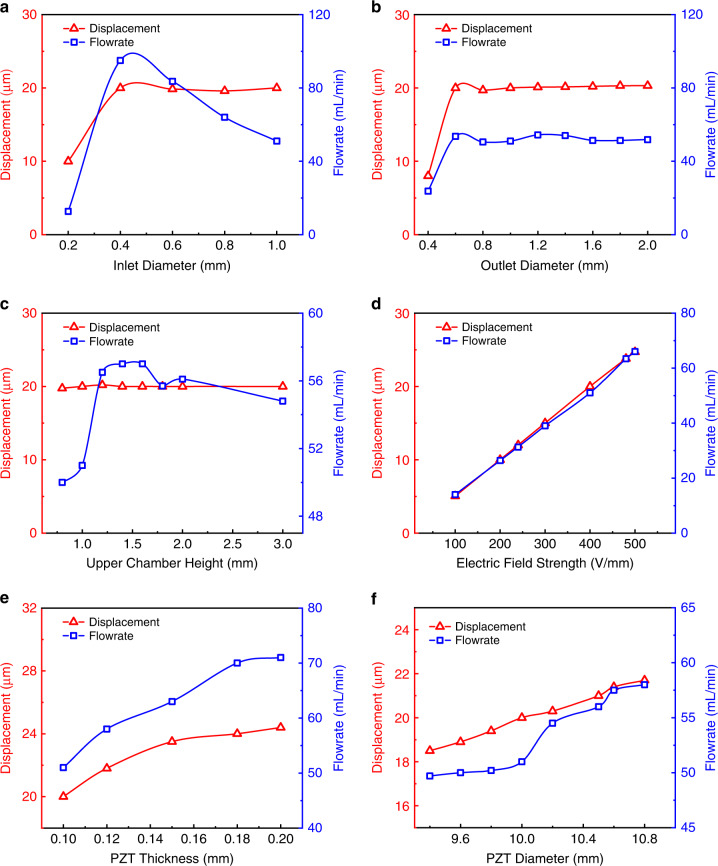
**Piezoelectric actuator displacement and output flowrate of the pump analyzed under different conditions.** **a** Diameter of the inlet (D1) from 0.2 to 1 mm. **b** Diameter of the outlet (D5) from 0.4 to 2 mm. **c** Height of the pump upper chamber (H1) from 0.8 to 3 mm. **d** Electric field strength from 100 to 500 V/mm. **e** Thickness of PZT (T2) from 0.1 to 0.2 mm. **f** Diameter of PZT (D3) from 9.4 to 10.8 mm

The structural parameters, such as the diameter of the inlet (D1) and outlet (D5) and the height of the pump upper chamber (H1), influence the output performance of the micropump, and they are investigated in detail. The displacement of the center of the PE actuator and air flowrate of the outlet are shown in Fig. 9a, b, and c, with the inlet and outlet diameters and height of the pump upper chamber serving as variables, respectively. As the diameter of the inlet increases from 0.2 to 1 mm, the central displacement of the actuator changes from 10 to 20 μm and stabilizes at 20 μm, while the gas flowrate increases from 12.7 to 95 mL/min and then decreases to 51 mL/min. This phenomenon occurs mainly because if the inlet diameter is too large, the efficiency of the fluid being sucked in is low, and the rate of backflow from the inlet increases^27^. As the outlet diameter increases from 0.4 to 2 mm, both the displacement and gas flowrate initially increase and then stabilize. The phenomenon changes with the change in the inlet diameter because of the structural differences below and above the PE actuator. Furthermore, the micropump performs poorly when both the inlet and outlet sizes are very small. Therefore, the performance of the pump is the best when the inlet and outlet diameters are 0.4 mm and 1.2 mm, respectively.

The upper chamber height has little influence on the displacement of the PE actuator because the space above the actuator is substantially larger than the amplitude of the actuator^28^. However, the gas flowrate increases from 50 to 57 mL/min and decreases to 54.8 mL/min when the upper chamber height increases from 0.8 to 1.3 mm (Fig. 9c). This variation is small and can be treated as a fluctuation of the numerical simulation. Therefore, the height of the upper chamber is selected to be 1.5 mm for further optimization.

The electric field strength applied on the piezoelectric ceramic affects the performance of the pump^29^, as shown in Fig. 9d. With increasing electric field strength, the displacement of the PE actuator and flowrate increase correspondingly. This phenomenon can be explained by Eq. (4). The induced stress is positively correlated to the electric field strength. However, if the electric field strength is more than 400–500 V/mm, the polarization strength of the piezoelectric ceramics may decrease over time (depolarization)^30,31^, and generate lots of heat. Therefore, a tradeoff between the output performance and driving electric field strength should be considered. In this work, the electric field strength is set as 400 V/mm.

4 $${S}_{j}={d}_{{ij}}{E}_{i}$$

where S_j_ is the stress; d_ij_ is the piezoelectric constant component; and E_i_ is the electric field strength.

The effects of the PZT plate thickness on the performance of the micropump are studied with the results shown in Fig. 9e. The displacement of the actuator and volume flowrate increase with the thickening of the PZT. However, to maintain a constant electric field strength on the PZT with increasing thickness, the applied voltage should increase correspondingly. However, a thicker PZT increases the resonant frequency of the system, which results in an increased flowrate. However, an increase in the working frequency contributes to heat. Moreover, the influence of the diameter of the PZT plate on the micropump is examined, as shown in Fig. 9f. The increase in the diameter of PZT boosts the performance of the micropump. These two phenomena can be explained by Eq. (4). Since the stress is linearly correlated to the electric field, with increasing PZT thickness, the total deformation of the PE actuator increases, and the overall volume change induced by the actuator increases, thereby improving the performance of the micropump. The diameter of the PZT plate is limited by that of the stainless steel, with a diameter of 11 mm. Within this size limitation, the larger the diameter of the PZT plate is, the better the performance of the micropump. Therefore, the size of PZT is selected to be 0.1 mm for the thickness and 10.8 mm for the diameter.

From the above analysis, the structural parameters of the micropump can be finalized properly. The above-analyzed parameters’ influences on the output performance of the micropump are summarized in Table 4, which shows the improvement in output performance of the PE micropump before and after optimization. The impact factors are ranked as shown in Table 4. The effect of the electric field strength on the output performance is the most significant.

**Table 4:** Influences of six parameters on the performance of the PE micropump

Parameters	Q_out-min_ (mL/min)	Q_out-max_ (mL/min)	Improvements	Rank
Inlet dia. (mm)	51	83.6	63.9%	2
Outlet dia. (mm)	50.5	54.4	7.7%	6
Upper chamber height (mm)	50	57	14%	5
Electric field intensity (V/mm)	14	66	312.5%	1
PZT thick. (mm)	51	71	39%	3
PZT dia. (mm)	49.7	58	16.7%	4

**Fig. 10:**
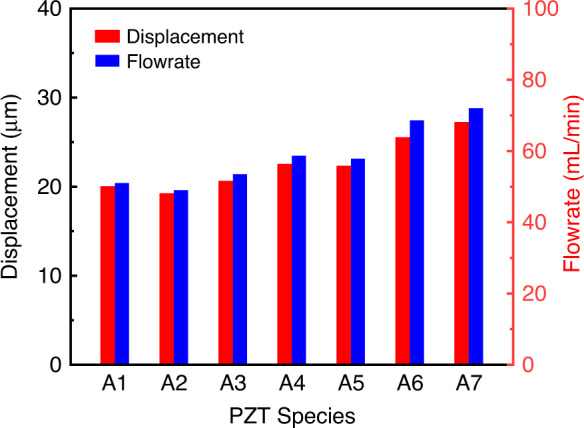
The performance of the micropump with different piezoelectric ceramics. PE actuator displacement and output gas flowrate under different piezoelectric ceramics

There are many species of PZT plates with different material properties, especially for piezoelectric constants. To assist in the selection of a suitable PZT plate for the fabrication of micropumps, it is necessary to investigate the effects of PZT plates with different material properties on the performance of the micropump. The PZT plates with different material parameters disclosed by Deci Technology (DECI PIEZO, Guangdong, China) are summarized in Table 5. The displacements of the actuator and the volume flowrate with different PZTs are shown in Fig. 10. From the analysis, PZT4 (A6) is selected as the piezoelectric material for the PE micropump in this work. Although the micropump using the A7 (PZT5) material has the best performance, its dielectric loss is too high, thus generating too much heat and deteriorating the performance of the micropump.

**Table 5:** Parameters of the piezoelectric ceramic

PZT Species			${\bf{A}}{\bf{1}}$	A2	A3	A4	A5	A6	A7
Density	$\rho$	$({10}^{3}{kg}/{m}^{3})$	7.5	7.6	7.6	7.6	7.8	7.8	7.9
Elastic constants	$s11$	$({10}^{-12}{m}^{2}/N)$	12.3	13.3	12.9	15	11.4	10.9	15.4
	$s12$		−4.1	−4.1	−4.1	−5.1	−3.5	−3.4	−4.8
	$s13$		−5.3	−6.9	−6.4	−9.3	−5.3	−4.8	−8.4
	$s33$		15.5	17.6	17.4	26.3	14.7	13.8	21.2
	$s44$		39	33.9	36.9	38.5	32.3	23.1	47.8
PE constants	${d}_{31}$	$({10}^{-12}C/N)$	−123	−125	−131	−135	−135	−150	−191
	${d}_{33}$		289	290	310	360	430	320	430
	${d}_{15}$		496	450	485	520	460	475	590
Relative permittivity	$\varepsilon 11$		1475	1460	1470	1530	1700	2530	1950
	$\varepsilon 33$		1300	1200	1350	1450	1650	2650	890
Dielectric loss	${\rm{\tan }}\delta$	(%)		0.5	0.8	0.8	0.5	0.5	1.5

**Fig. 11:**
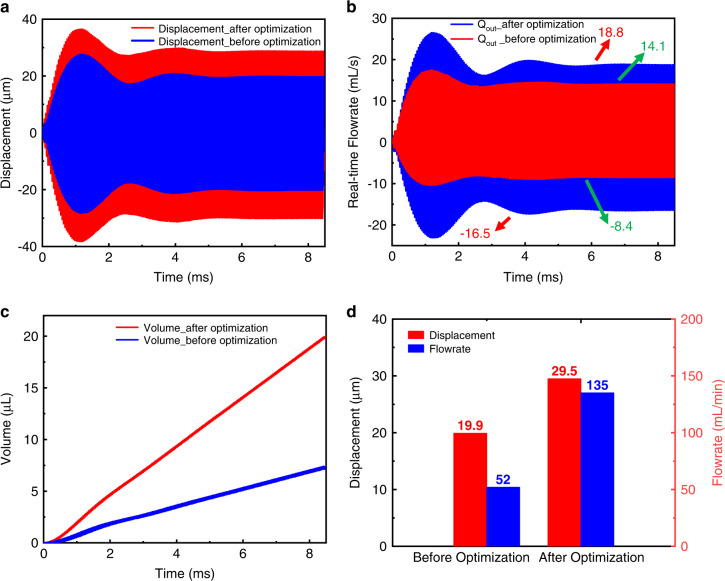
Performance comparison before and after optimization of the micropump. **a** displacement of the center point of the PE actuator. **b** real-time gas volume flowrate in the outlet. **c** gas volume flow out of the outlet of the pump with time, and **d** comparison of displacement and gas volume flowrate before and after optimization

Figure 11 shows a comparison of the performance characteristics of the micropumps before and after optimization. With the optimized structure and optimal piezoelectric ceramics selected, the final performance is significantly improved. The displacement is increased by 48.2%, and the output gas flowrate is increased by 159.6%, reaching 135 mL/min, relative to those without optimization.

**Fig. 12:**
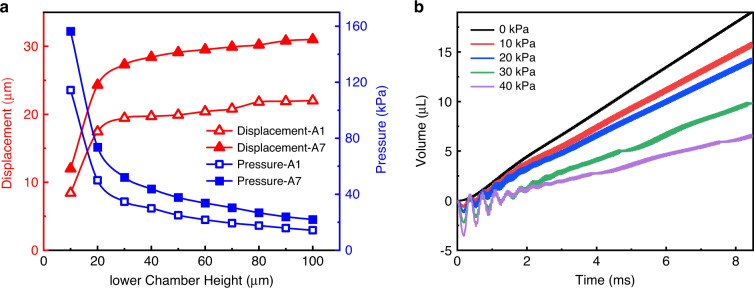
Simulation analysis of the micropump. **a** PE actuator displacement and maximum pressure in the pump chamber as a function of the height of the lower chamber (H2). **b** Simulated gas volume outputs of the micropump under different pressure loads with time

The displacement amplitudes change when different piezoelectric plates are used. Therefore, the height of the lower chamber is further optimized. The simulations are performed using materials A1 and A7 as the piezoelectric plate. The simulation results are shown in Fig. 12a. Clearly, the displacements of both the piezoelectric plates are very small when the height of the lower chamber is very small. As the height increases from 10 to 100 μm, the central displacement of the actuator gradually increases and stabilizes, while the variation in the maximum pressure in the chamber with the lower chamber height exhibits the opposite phenomenon. The optimal height of the lower chamber for the A1 plate is approximately 20 μm, while that for the A7 plate is approximately 30 μm, which is closely related to the maximum displacement of the center of the actuator.

For gas micropumps, in addition to the gas flowrate, the maximum achievable pressure is a very important parameter. In this work, various pressure loads are applied at the outlet to investigate the gas flow performance of the micropump, with the results shown in Fig. 12b. Obviously, as the pressure load increases, the volume of gas pumped out per unit time decreases. The micropump can push gas out even under a 40 kPa pressure load, and the flowrate reaches 46 mL/min.

## Experimental results

**Fig. 13:**
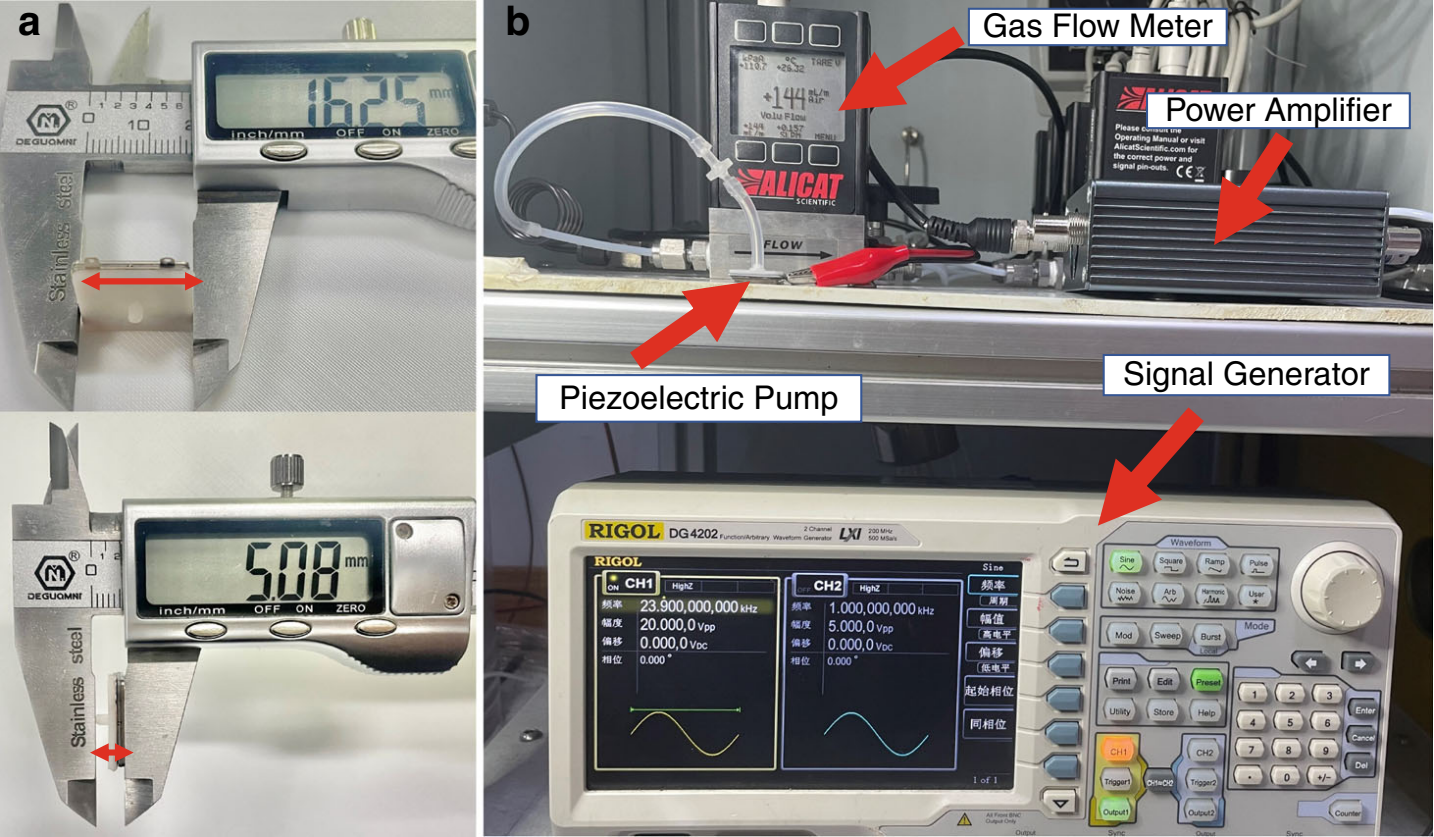
Experimental equipment. **a** Photographs and dimensions of the fabricated micropump. **b** Test setup for characterizing the PE micropump

Based on the above numerical analysis, a micropump was designed and fabricated. The PE micropump was assembled using a low-temperature curing epoxy resin glue. The glue was applied through a high-mesh screen printing technique and was followed by heating and bonding processes. The fabricated micropump is shown in Fig. 13a.

The performance of the fabricated micropump was characterized using the following equipment. The impedance of the micropump was measured using an impedance analyzer (E4990A-120, Keysight, USA), and the displacement of the PE actuator central point of the micropump was measured using a laser scanning vibrometer (LV-S01-M, SUNNY Optical Intelligence, China). The flowrate performance was measured by a high-precision gas flowmeter (Alicat, USA). The experimental setup is shown in Fig. 13b.

The excitation electrical signal was generated by a signal generator (DG4204, RIGOL, China) and amplified by a power amplifier. For the measurement under an excitation signal with a peak voltage of 40 V, the signal frequency was swept from 20 to 30 kHz in a step of 100 Hz. The measured displacement and impedance of the PE actuator are shown in Fig. 15a with a maximum displacement at a frequency of 23800 Hz. This value is only 158 Hz higher than the simulated one, implying high accuracy of the developed model.

**Fig. 14:**
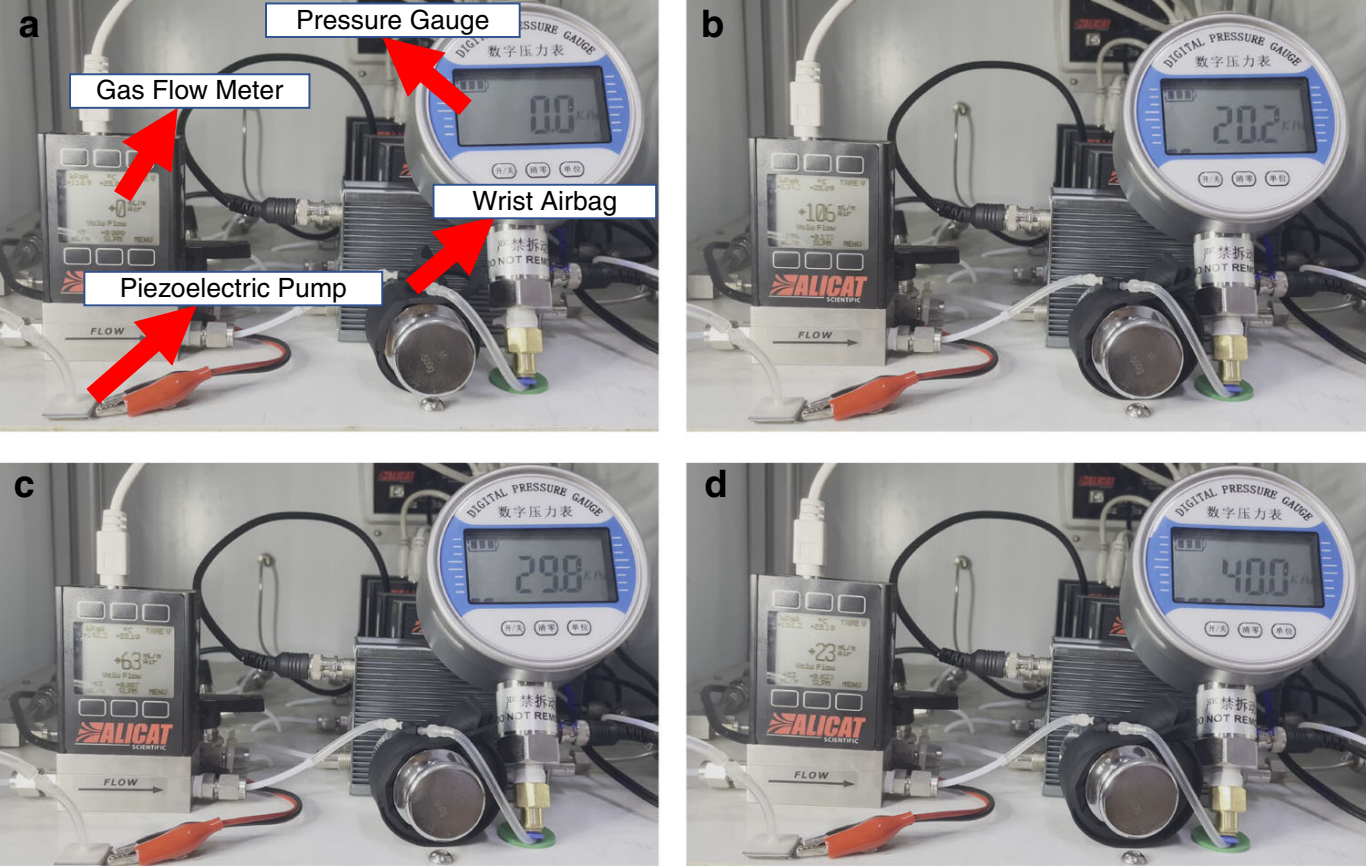
Real-time flowrate under various pressure loads. **a** 0 kPa. **b** 20.2 kPa. **c** 29.8 kPa and **d** 40 kPa

**Fig. 15:**
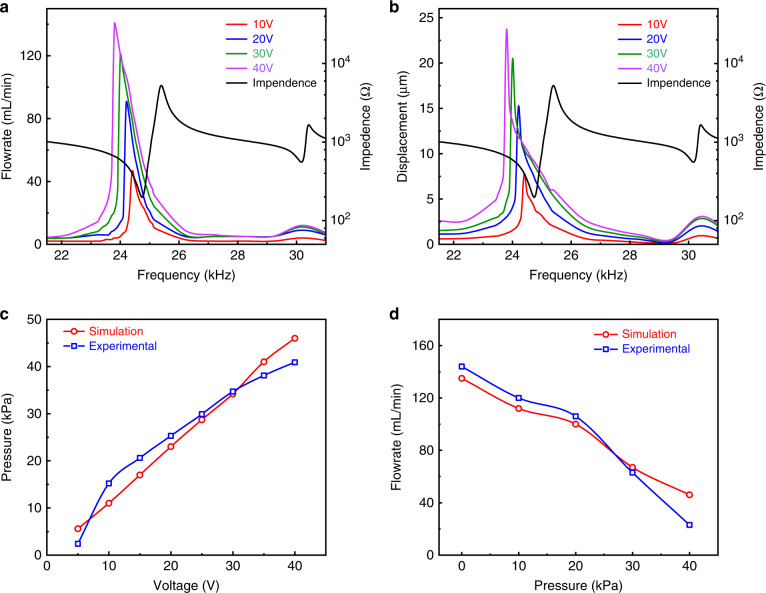
Experimental analysis of the micropump. **a** Gas flowrate of the micropump at different drive voltage amplitudes and frequencies. **b** Displacement of the center point of the PE actuator at different drive voltage amplitudes and frequencies. **c** Maximum pressure of the micropump achieved under various driving voltage amplitudes. **d** Gas flowrate of the micropump under various pressure loads

Then, an airbag and a digital pressure gauge were used to characterize the micropump performance under different pressure loads. The measurement setup and performance of the pump with pressure load are shown in Fig. 14. The output of the flowmeter was connected to one port of the airbag, and the other port of the airbag was connected to the pressure gauge. The flowrate and the load pressure were recorded in real-time, and the flowrates under various pressure loads were obtained. The mass flow meter and the pressure gauge were calibrated before the experiment, and the measurement environment involved a room temperature of 20 °C and atmospheric pressure of 101.3 kPa. Clearly, when the micropump worked at a pressure load of 40 kPa, it maintained an output flowrate of 23 mL/min, which was in agreement with the simulated result. By comparing the simulated results with the experimental results, the measured output flowrate was larger than the simulated output flow rate at low pressure, but it was smaller than the simulated output flowrate at high pressure. Nevertheless, the flowrates were highly consistent, as shown in Fig. 15d.

The flowrate of the pump and displacement of the PE actuator under various driving voltages and frequencies are shown in Fig. 15a, b, respectively. The flowrate increased with increasing signal amplitude, but the operation frequency decreased accordingly. The same trends were observed for the impedance of the PE actuator. The maximum pressure under various driving signal amplitudes is shown in Fig. 15c, displaying a linear relationship. Moreover, as shown in Fig. 15d, as the pressure load increased, the gas flowrate decreased monotonically, maintaining a value of 20 mL/min even under a 40 kPa pressure load.

There are many application scenarios for this type of high flowrate and pressure load pump, such as wearable blood pressure measurement devices. For blood pressure measurement, the maximum pressure produced by the pump must be higher than the maximum blood pressure of humans, which is usually approximately 250 mmHg (33.3 kPa)^32^. Therefore, the pressure (maximum 40 kPa) produced by our micropump should be able to stop the blood flow in the blood vessel. In addition to the pressure load, the flowrate of the micropump is an important parameter. The flowrate determines the time needed to pump up the airbag for the blood pressure measurement. For a wearable flexible airbag, which is worn on the wrist, its volume is approximately 20 mL (10 cm *2 cm *1 cm). It would take approximately 35 seconds to pump the airbag to 40 kPa, which is acceptable. In real blood pressure application scenarios, the flowrate should be controlled by a controller that is dependent on the algorithm of the blood pressure measurement device. A video of the real-time performance of the micropump pumping a wearable airbag can be seen in the Supplemental Information.

## Conclusion

In this work, the analysis, design, and fabrication of the PE micropump were demonstrated. A 3D simulation model of the PE micropump was established, and bidirectional fluid–structure coupling analysis was carried out to obtain the flow velocity and direction of the gas in the pump chamber. Then, the parameters affecting the micropump output performance were analyzed and optimized. After optimization, the output flowrate of the PE micropump was 114.2 mL/min, and the flowrate was improved by 119.6% relative to the structure without optimization. Seven different species of PZTs with different material parameters were used to analyze their effects on the micropump, and the suitable PZT material was selected. Combined with the optimized pump structure and PZT material, the flowrate output performance of the PE micropump finally reached 135 mL/min, which is 159.6% higher than that of the unoptimized micropump. Then, the gas flow characteristics of the micropump under various pressure loads were investigated, and the results showed that the pump could still deliver gas directionally and stably under a 40 kPa pressure load. Based on the analysis, the micropump was fabricated and assembled. The measurement results showed that when the micropump worked at the first-order resonance frequency of 23.9 kHz, with a driving sine signal amplitude of 40 V, the output flowrate reached the maximum value of 144 mL/min. When measuring with different load pressures, the micropump still had an output flowrate of 23 mL/min under a pressure load of 40 kPa, which was consistent with the FEM analysis results.

## Supplementary information

Supplement Informtion

## References

[1] Kaçar A, Özer MB, Taşcıoğlu Y (2020). A Novel Artificial Pancreas: Energy Efficient Valveless Piezoelectric Actuated Closed-Loop Insulin Pump for T1DM. Appl. Sci..

[2] Fu YQ (2017). Advances in piezoelectric thin films for acoustic biosensors, acoustofluidics and lab-on-chip applications. Prog. Mater. Sci..

[3] Bußmann A (2021). Piezoelectric Silicon Micropump for Drug Delivery Applications. Appl. Sci..

[4] Liu C, Zhu Y, Wu C (2020). Optimization of a synthetic jet based piezoelectric air pump and its application in electronic cooling. Microsyst. Technol..

[5] He L (2019). Exploration on relationship between flowrate and sound pressure level of piezoelectric pump. Microsyst. Technol..

[6] Jiang H (2022). A flowrate on-line monitoring method for piezoelectric pump based on self-sensing circuit. J. Mech. Sci. Technol..

[7] Jiang W (2020). Efficient bidirectional piezo-optomechanical transduction between microwave and optical frequency. Nat. Commun..

[8] Wu Y (2013). An improved resonantly driven piezoelectric gas pump. J. Mech. Sci. Technol..

[9] Dong JS (2016). Design and experimental research on piezoelectric pump with triple vibrators. Microsyst. Technol..

[10] Liu C, Zhu Y (2019). Simulation and experimental study of direct spray type piezoelectric air pumps based on synthetic jet. Microsyst. Technol..

[11] Asadi Dereshgi H, Dal H, Yildiz MZ (2021). Piezoelectric micropumps: state of the art review. Microsyst. Technol..

[12] Aboubakri A, Ahmadi VE, Koşar A (2020). Modeling of a Passive-Valve Piezoelectric Micro-Pump: A Parametric Study. Micromachines.

[13] Yeming S, Junyao W (2018). Digitally-controlled driving power supply for dual-active-valve piezoelectric pump. Microsyst. Technol..

[14] Zeng P (2015). Structure design and experimental study on single-bimorph double-acting check-valve piezoelectric pump. Proc. Inst. Mech. Eng., Part C: J. Mech. Eng. Sci..

[15] Guan Y (2019). Performance Analysis of a Microfluidic Pump Based on Combined Actuation of the Piezoelectric Effect and Liquid Crystal Backflow Effect. Micromachines (Basel).

[16] Izzo I, Accoto D, Menciassi A, Schmitt L, Dario P (2007). Modeling and experimental validation of a piezoelectric micropump with novel no-moving-part valves. Sens. Actuators A: Phys..

[17] Zhang J, Wang Y, Huang J (2018). Equivalent Circuit Modeling for a Valveless Piezoelectric Pump. Sens. (Basel).

[18] Ji J (2019). Design and Experimental Verification on Characteristics of Valve-Less Piezoelectric Pump Effected by Valve Hole Spacing. IEEE Access.

[19] Li H, Liu J, Li K, Liu Y (2021). A review of recent studies on piezoelectric pumps and their applications. Mech. Syst. Signal Process..

[20] Guan Y, Bai M, Meng X, Liu Y, Xu F (2020). Experimental Investigation of Piezoelectric Micropumps with Single, Series or Parallel Pump Chambers. Int. J. Acoust. Vib..

[21] Wang XY, Ma YT, Yan GY, Huang D, Feng ZH (2014). High flow-rate piezoelectric micropump with two fixed ends polydimethylsiloxane valves and compressible spaces. Sens. Actuators A: Phys..

[22] Ghaffari O, Solovitz SA, Ikhlaq M, Arik M (2016). An investigation into flow and heat transfer of an ultrasonic micro-blower device for electronics cooling applications. Appl. Therm. Eng..

[23] Reif, B. P. & Durbin, P. *Statistical theory and modeling for turbulent flows*. (John Wiley & Sons, 2011).

[24] Pope, S. B. & Pope, S. B. *Turbulent flows*. (Cambridge university press, 2000).

[25] Hanjalić K, Launder BE (1972). A Reynolds stress model of turbulence and its application to thin shear flows. J. fluid Mech..

[26] Van LL (2020). Simulation and Experimental Study of a Synthetic Jet Valveless Pump. IEEE/ASME Trans. Mechatron..

[27] Dong J (2020). Design and experimental research of piezoelectric pump based on macro fiber composite. Sens. Actuators A: Phys..

[28] Ji J (2021). A serial piezoelectric gas pump with variable chamber height. Sens. Actuators A: Phys..

[29] Li H, Liu J, Feng Y, Deng J, Liu Y (2022). A Broadband, High-Power Resonant Piezoelectric Active-Valve Pump Driven by Sandwich Bending Transducers. IEEE Trans. Ind. Electron.

[30] Li F-X, Fang D-N (2005). Effects of electrical boundary conditions and poling approaches on the mechanical depolarization behavior of PZT ceramics. Acta Materialia.

[31] Fialka J (2016). Measurement of thermal depolarization effects in piezoelectric coefficients of soft PZT ceramics via the frequency and direct methods. J. Eur. Ceram. Soc..

[32] Rothwell PM (2010). Prognostic significance of visit-to-visit variability, maximum systolic blood pressure, and episodic hypertension. Lancet.

